# Infant Antibody Repertoires during the First Two Years of Influenza Vaccination

**DOI:** 10.1128/mbio.02546-22

**Published:** 2022-10-31

**Authors:** Masayuki Kuraoka, Nicholas C. Curtis, Akiko Watanabe, Hidetaka Tanno, Seungmin Shin, Kevin Ye, Elizabeth Macdonald, Olivia Lavidor, Susan Kong, Tarra Von Holle, Ian Windsor, Gregory C. Ippolito, George Georgiou, Emmanuel B. Walter, Garnett Kelsoe, Stephen C. Harrison, M. Anthony Moody, Goran Bajic, Jiwon Lee

**Affiliations:** a Department of Immunology, Duke University grid.471396.egrid.26009.3dgrid.471396.e , Durham, North Carolina, USA; b Thayer School of Engineering, Dartmouth College, Hanover, New Hampshire, USA; c Department of Chemical Engineering, University of Texas, Austin, Texas, USA; d Department of Molecular Biosciences, University of Texas, Austin, Texas, USA; e Department of Biomedical Engineering, University of Texas, Austin, Texas, USA; f Institute for Cellular and Molecular Biology, University of Texas, Austin, Texas, USA; g Laboratory of Molecular Medicine, Boston Children’s Hospital and Harvard Medical School, Boston, Massachusetts, USA; h Department of Pediatrics, Duke University grid.471396.egrid.26009.3dgrid.471396.e , Durham, North Carolina, USA; i Duke Human Vaccine Institute, Duke University grid.471396.egrid.26009.3dgrid.471396.e , Durham, North Carolina, USA; j Department of Surgery, Duke University, Durham, North Carolina, USA; k Howard Hughes Medical Institute, Harvard Medical School, Boston, Massachusetts, USA; l Department of Microbiology, Icahn School of Medicine at Mount Sinai, New York, New York, USA; GSK Vaccines

**Keywords:** B cell memory, circulating antibodies, immune imprinting, influenza virus, viral immunity

## Abstract

The first encounter with influenza virus biases later immune responses. This “immune imprinting,” formerly from infection within a few years of birth, is in the United States now largely from immunization with a quadrivalent, split vaccine (IIV4 [quadrivalent inactivated influenza vaccine]). In a pilot study of IIV4 imprinting, we used single-cell cultures, next-generation sequencing, and plasma antibody proteomics to characterize the primary antibody responses to influenza in two infants during their first 2 years of seasonal influenza vaccination. One infant, who received only a single vaccination in year 1, contracted an influenza B virus (IBV) infection between the 2 years, allowing us to compare imprinting by infection and vaccination. That infant had a shift in hemagglutinin (HA)-reactive B cell specificity from largely influenza A virus (IAV) specific in year 1 to IBV specific in year 2, both before and after the year 2 vaccination. HA-reactive B cells from the other infant maintained a more evenly distributed specificity. In year 2, class-switched HA-specific B cell *IGHV* somatic hypermutation (SHM) levels reached the average levels seen in adults. The HA-reactive plasma antibody repertoires of both infants comprised a relatively small number of antibody clonotypes, with one or two very abundant clonotypes. Thus, after the year 2 boost, both infants had overall B cell profiles that resembled those of adult controls.

## INTRODUCTION

Influenza A viruses (IAV) and influenza B viruses (IBV) that circulate in the human population evolve in response to acquired global host immunity. Antigenic drift, from mutation and selection for resistance against existing immunity, generates a new “antigenic cluster” every 5 to 10 years, depending on the influenza type and subtype (1). Most of these variations occur within the hemagglutinin (HA) protein.

Classic epidemiological and serological studies, as well as contemporary molecular analyses, indicate that the antigenic variant of an influenza virus subtype first encountered conditions later responses—a phenomenon called “original antigenic sin” in early papers and now known as “immune imprinting” (2–5). The presumed mechanism is that immune memory from the earliest exposure, both in the form of memory B cells (Bmem) and long-lived plasma cells (LLPCs), which secrete and maintain steady-state plasma antibodies, governs the immune outcome of all subsequent exposures. In accord with such a mechanism, lineage reconstructions have shown that B cells elicited by vaccination of adults are descendants of those elicited by the presumptive infecting strain circulating at the time of the donors’ birth (6). Less clear are the extent to which the subtype of the initial exposure dominates the response to multivalent vaccines and whether the B cell memory established by exposure to a new subtype (e.g., H3 in 1968 for those imprinted earlier by H1) is primarily a naive response or rather modification or adaptation of Bmem from the initial imprinting. The usually uncertain history of various exposures to IAV and IBV between early childhood and sample collection has confounded the interpretation of most data from individual adult donors. Because the only definitively primary responses are in previously uninfected infants and young children receiving their first vaccination, we have initiated an effort to resolve some of these ambiguities with the pilot study reported here.

Analyses of immune responses in infants require methods that can derive sufficient information from the small blood samples that can be drawn ethically. While previous systems immunology efforts have examined the immune system of infants, for example, by use of cytometry by time of flight (7), they did not generate information about the specificities of the elicited cellular and humoral responses. In this study, we analyzed the cellular and serological influenza virus antibody repertoires of two infants after their first quadrivalent inactivated influenza vaccine (IIV4) immunization and followed the development of HA-specific immune responses over 2 years. One of the two infants, who received a prime but not a boost in year 1, had a documented IBV infection between sample collection in year 1 and year 2, providing an indirect comparison of the responses to vaccination and infection. In this infant, there was a shift after the infection from an IAV- to an IBV-dominated Bmem repertoire. The other infant, who received both prime and boost in year 1, had more balanced responses. In both infants, year 2 somatic hypermutation (SHM) levels approached those observed in adults. The results suggest that IIV4 may provide a less subtype-dominant, although potentially weaker imprint than does (monovalent) infection. A more definitive conclusion will require obtaining and analyzing samples from more than the two subjects in this initial pilot study.

## RESULTS

### Study design.

**FIG 1:**
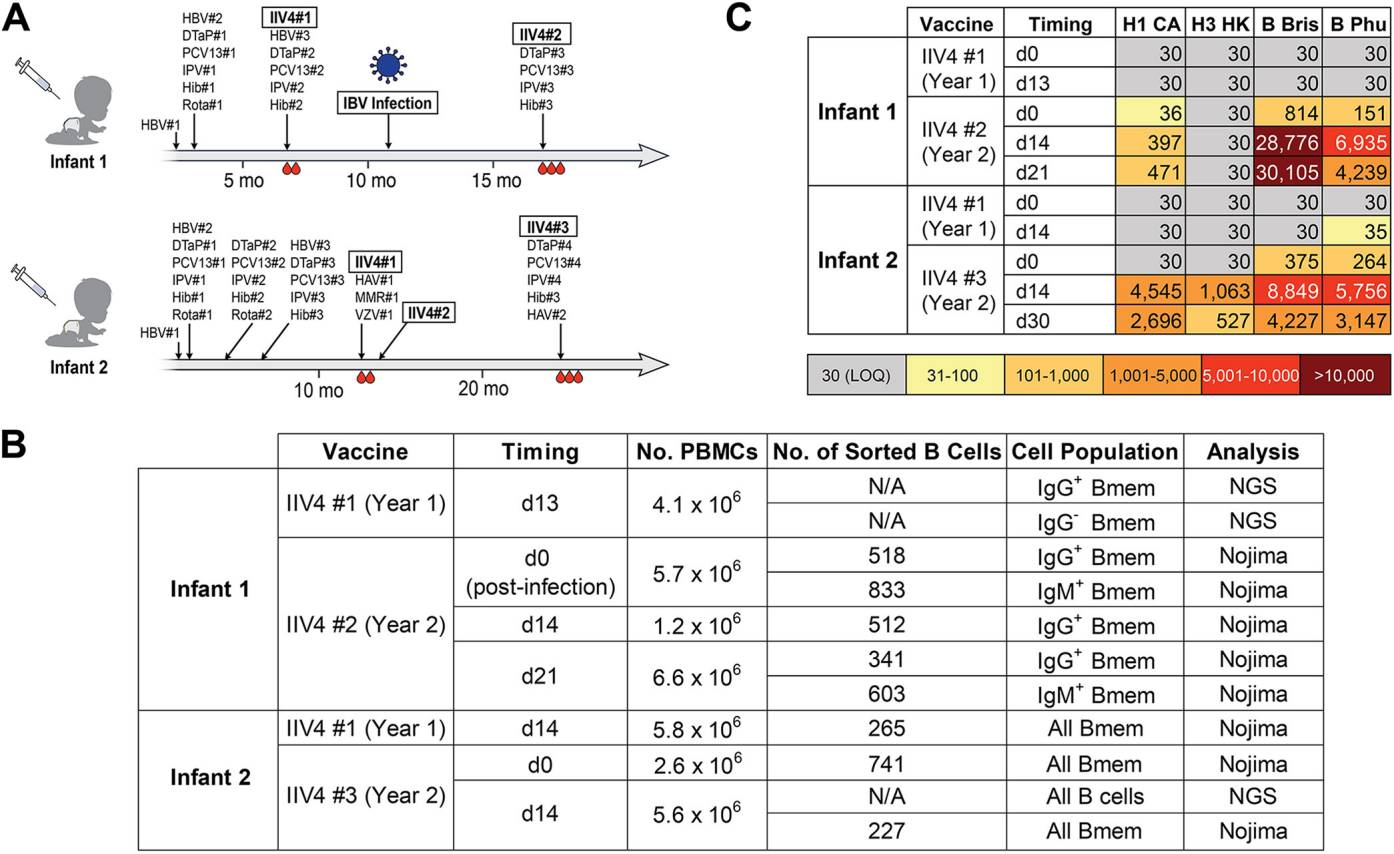
Study design and sample description. (A) Immunization and influenza infection history for the two infants enrolled in the study. Blood drop symbols indicate the blood draws analyzed in the study. HBV, hepatitis B vaccine; DTaP, diphtheria, tetanus, and pertussis vaccine; PCV, pneumococcal vaccine; IPV, polio vaccine; Hib, Haemophilus influenzae type B vaccine; Rota, rotavirus vaccine; IIV4, quadrivalent inactivated influenza vaccine; HAV, hepatitis A virus vaccine; MMR, measles, mumps, and rubella vaccine; VZV, varicella-zoster virus vaccine. (B) Summary of B cells analyzed in this study. (C) Plasma IgG binding titers against HAs included in the IIV4. Values indicate ED_50_s from ELISA. H1 CA, A/California/2009 X181; H3 HK, A/Hong Kong/2014 X263B; B Bris, B/Brisbane/60/2008; B Phu, B/Phuket/3073/2013; LOQ, limit of quantitation.

We enrolled two infants, designated infant 1 and infant 2, with exposure histories shown in Fig. 1A. Both infants received IIV4 concurrently with other vaccines during their first 2 years (Table S1 in the supplemental material). Infant 1 received only a prime IIV4 in year 1 and contracted a confirmed, adventitious IBV infection between the year 1 and year 2 IIV4 vaccinations. Yamagata lineage IBVs were more prevalent than Victoria lineage IBVs both throughout the influenza season in question (2016–2017) and during the week of diagnosis (https://www.cdc.gov/flu/weekly/index.htm). Thus, the infecting virus was likely a Yamagata lineage IBV, but as both lineages were circulating, we cannot be certain. Infant 2 followed the standard IIV4 vaccination schedule, with a prime and a boost in year 1. Blood samples were collected at pre- and postvaccination time points as indicated in Fig. 1A and B.

10.1128/mbio.02546-22.5 TABLE S1 IIV immunizations and their components. Download Table S1, PDF file, 0.05 MB. Copyright © 2022 Kuraoka et al. 2022 Kuraoka et al. https://creativecommons.org/licenses/by/4.0/ This content is distributed under the terms of the Creative Commons Attribution 4.0 International license.

Although the different sampling histories for the two infants studied here diverged in year 1, with the lack of a booster vaccination for infant 1, they converged in year 2, with the “boost” for infant 1 being the intervening IBV infection. Thus, by the time of the year 2 vaccination, both subjects had two exposures: in one case, a primary vaccination was followed several months later by infection, and in the other, primary vaccination was followed 1 month later by a second, identical boost vaccination. All the vaccinations were with split vaccines—i.e., protein antigens; the infection was an “immunization” with intact virions. The two infants were a year apart in age and, hence, in immunization schedules. The 2016–2017 and 2017–2018 influenza vaccines differed only in their H1N1 components; the 2017–2018 and 2018–2019 vaccines differed in their H3N2 components and in their Victoria lineage IBV components.

We measured the HA-binding titers of plasma IgG at each time point by enzyme-linked immunosorbent assay (ELISA) (Fig. 1C). In year 1, both infants had titers below the limit of quantitation for any of the IIV4 HA components. In year 2, infant 1 had a marked increase in IgG titers for IBV HAs and substantially lower titers for IAV HAs (Fig. 1C), while infant 2 had similar titers for both.

We investigated the repertoires of both circulating antibody and HA-specific memory B cells (Bmem). For cellular responses, we profiled the B cells collected from each time point by next-generation sequencing (NGS) and by clonal expansion in Nojima cultures and sequencing (Fig. 1B) (8). To analyze plasma antibody repertoires, we isolated plasma IgG from the year 2 postvaccination samples only, as there were insufficient amounts of HA-reactive IgG in plasma samples collected in year 1. We identified the sequences and quantities of each HA-reactive plasma IgG clonotype by combining high-throughput B cell sequencing with high mass-resolution proteomics, as in our previous work (9–12).

### Analysis of the overall B cell repertoire.

**FIG 2:**
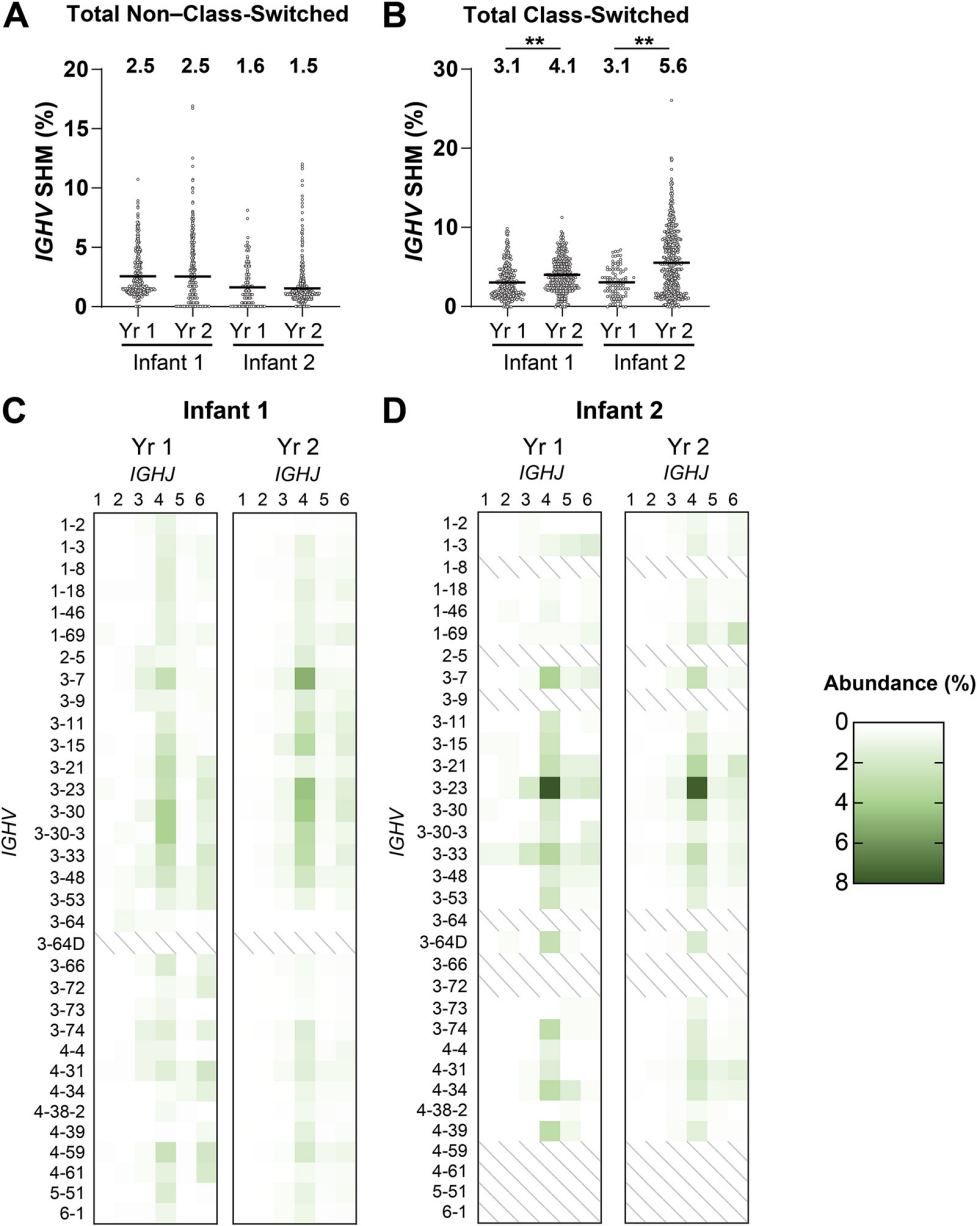
Total B cell repertoire. (A and B) The *IGHV* SHM levels of non–class-switched (A) and class-switched (B) total postvaccination B cells. Median values are represented by horizontal lines, and mean values are included above each data set. Statistical significance was determined by Mann-Whitney U test (**, *P < *0.01). (C and D) *IGHV-IGHJ* usage of postvaccination B cells across 2 years for infant 1 (C) and infant 2 (D). *IGHV* with >1% abundance in either infant was included in the data set.

Affinity maturation, a consequence of SHM and antigen-driven selection, and antibody class switching are hallmarks of the B cell response to protein antigens. We found no significant increase in the *IGHV* SHM levels for all non–class-switched B cells in the two infants from year 1 to year 2 (Fig. 2A). The average SHM levels for non–class-switched B cells at all time points (~1.5% to 2.5%) were similar to those found in adults (13–15), in accord with a published study (16). For all class-switched B cells, the average *IGHV* SHM levels in both infants increased significantly from year 1 to year 2 (Fig. 2B), more prominently in infant 2 (from 3.1% to 5.6%) than in infant 1 (from 3.1% to 4.1%). The difference might be due to the greater number of vaccinations (28 for infant 2 and 18 for infant 1) that infant 2 received during the time period represented here (Fig. 1A); infant 2 was also ~6 months older than infant 1 at comparable times of blood draw.

B cell receptors (BCRs) from Bmem had similar overall *IGHV* distributions in both children (Fig. 2C and D), with some prevalence of *IGHV 3-7*, *IGHV 3-23*, and *IGHV 3-33*; *IGHJ4* was the most abundant J segment. Other molecular features, such as the length, hydrophobicity, and charge of the complementarity-determining region 3 of the immunoglobulin heavy chain (CDR-H3), which can influence antibody engagement with a variety of different antigens, did not differ significantly between the two infants or among time points (Fig. S1) and were similar to the corresponding characteristics observed in adults (17).

10.1128/mbio.02546-22.1 FIG S1 CDR-H3 length, charge, and hydrophobicity of total postvaccination B cells. Median values are represented by horizontal lines, and mean values are included above each dataset. Download FIG S1, PDF file, 0.6 MB. Copyright © 2022 Kuraoka et al. 2022 Kuraoka et al. https://creativecommons.org/licenses/by/4.0/ This content is distributed under the terms of the Creative Commons Attribution 4.0 International license.

### Binding profiles of HA-specific memory B cells.

We characterized the binding of HA-specific Bmem at each time point by antigen-independent single-cell sorting of Bmem from peripheral blood mononuclear cell (PBMC) samples (Fig. S2) into Nojima culture wells (8). We then profiled the binding specificities and avidities of the secreted antibodies using a panel of IAV and IBV HAs in a multiplexed bead assay (Data Set S1 and Fig. S3) (18, 19). We determined the HA specificity and binding avidity by calculating an avidity index (AvIn) for each antibody, as previously described (8). Because Bmem collected from infant 1 in year 1 was analyzed only by NGS without the Nojima culture step and the ensuing multiplexed bead assay, we expressed representative recombinant antibodies selected based on the highest read counts and evaluated their antigen-binding properties by ELISA (Table S2).

10.1128/mbio.02546-22.2 FIG S2 Representative sorting strategy to isolate Bmem for Nojima culturing. Download FIG S2, PDF file, 0.08 MB. Copyright © 2022 Kuraoka et al. 2022 Kuraoka et al. https://creativecommons.org/licenses/by/4.0/ This content is distributed under the terms of the Creative Commons Attribution 4.0 International license.

10.1128/mbio.02546-22.3 FIG S3 Luminex reactivity assays. Red lines indicate background measurements for each antigen. List on the right shows antigen corresponding to each numbered column. See Materials and Methods, “Multiplex bead assay,” for full name of each HA and control antigen. IgG, mouse anti-human IgG. Antigens marked H are trimeric, head-only constructs. Download FIG S3, PDF file, 0.5 MB. Copyright © 2022 Kuraoka et al. 2022 Kuraoka et al. https://creativecommons.org/licenses/by/4.0/ This content is distributed under the terms of the Creative Commons Attribution 4.0 International license.

10.1128/mbio.02546-22.6 TABLE S2 Recombinant MAbs from Bmem of infant 1 year 1 postvaccination. Clonotypes with high read counts from the NGS data were made as MAbs. For each selected clonotype, the CDR-H3 sequence, *IGHV*, *IGHJ*, and SHM levels are shown. MAbs were characterized by ELISA to determine their EC_50_ values against HAs. +++, <100 nM; ++, 100 to 1,000 nM; +, detected signal at 1,000 nM; −, no signal at 1,000 nM; H1, A/California/2009 X181; H3, A/Hong Kong/2014 X263B; B/Vic, B/Brisbane/60/2008; B/Yama, B/Phuket/3073/2013. Download Table S2, PDF file, 0.05 MB. Copyright © 2022 Kuraoka et al. 2022 Kuraoka et al. https://creativecommons.org/licenses/by/4.0/ This content is distributed under the terms of the Creative Commons Attribution 4.0 International license.

10.1128/mbio.02546-22.9 DATA SET S1 Sequences and molecular features of individual HA-reactive Bmem and corresponding Luminex reactivity data. In column under “Head construct,” “Yes” indicates that the IgG bound *any* of the head-only constructs in the Luminex panel. For full sequences, see “Data availability”. Download Data Set S1, XLSX file, 0.02 MB. Copyright © 2022 Kuraoka et al. 2022 Kuraoka et al. https://creativecommons.org/licenses/by/4.0/ This content is distributed under the terms of the Creative Commons Attribution 4.0 International license.

**FIG 3:**
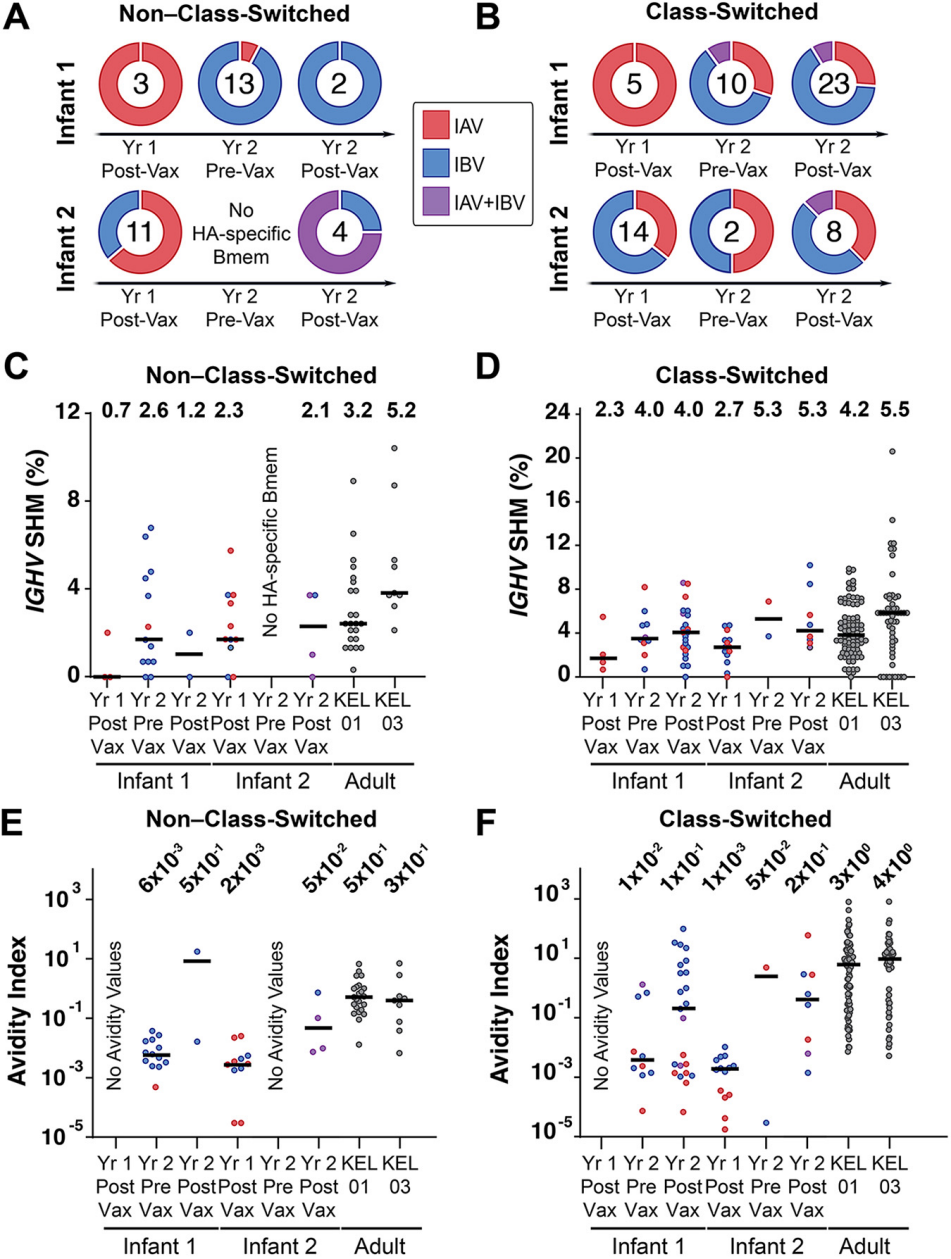
Features of HA-specific Bmem. (A and B) Binding specificities of HA-specific non–class-switched (A) and class-switched (B) Bmem. Doughnut plots represent percentages of Bmem that were IAV specific, IBV specific, or cross-reactive to both (IAV+IBV). Numbers indicate sample size (number of HA-reactive Bmem) at each time point. (C and D) *IGHV* SHM levels of HA-specific non–class-switched (C) and class-switched (D) Bmem. (E and F) Avidity index values of HA-specific non–class-switched (E) and class-switched (F) Bmem. For panels C to F, median values are represented by horizontal lines, and mean (C and D) or geometric mean (E and F) values are included above each data set. Two adult subjects studied previously are designated KEL01 (male, age 39) and KEL03 (female, age 39). Red, blue, and purple dots represent IAV-specific, IBV-specific, and IAV+IBV Bmem, respectively.

We assigned the binding specificities of non–class-switched (Fig. 3A) and class-switched (Fig. 3B) Bmem as IAV specific or IBV specific based on their relative AvIn values (8). A small population of Bmem bound both IAV and IBV HAs with similar, usually low, AvIn values, and we designated them as IAV+IBV cross-reactive. The sampled response of infant 1 shifted from entirely IAV HA specific (non-class switched, IAV HA specific/Total HA specific = 3/3; class switched, 5/5) in year 1 postvaccination (after IIV4 no. 1) to largely IBV HA specific (non-class switched, IBV HA specific/Total HA specific = 12/13; class switched, 6/10) in year 2 prevaccination (before IIV4 no. 2), the earliest sample time after the IBV infection. The lineage (Yamagata or Victoria) of that infection was not determined. The IBV-biased outcome continued after the year 2 IIV4 no. 2 immunization (non-class switched, IBV HA specific/Total HA specific = 2/2; class switched, 15/23). We conclude that the IBV infection caused the shift in Bmem reactivity. For infant 2, the binding specificities were more evenly distributed at each sampling time (Fig. 3A and B).

The mean AvIn values of both non–class-switched and class-switched Bmem (Fig. 3E and F) increased across time points, as expected from affinity maturation of HA-specific Bmem. In infant 1, most of the high-avidity Bmem were IBV specific, and the (geometric) mean and median AvIn values of all the IBV-specific, year 2 postvaccination Bmem (~1 × 10^0^ and ~3 × 10^0^, respectively) in that infant (Data Set S1) were indistinguishable from those of the control adults, two previously studied subjects reporting repeated seasonal influenza vaccination (Fig. 3F) (18). Infant 2 had more evenly distributed binding specificities and avidities after the year 2 vaccination.

We analyzed postvaccination SHM levels for HA-specific Bmem in both years. The class-switched, HA-specific Bmem SHM values increased from 2.3% and 2.7% in year 1 for infant 1 and infant 2, respectively, to 4.0% and 5.5% in year 2, with essentially no change in SHM levels for non–class-switched Bmem (0.7% to 1.2% for infant 1 and 2.3% to 2.1% for infant 2) (Fig. 3C and D). Adult controls had 3.2% and 5.2% average SHM levels for non–class-switched and 4.2% and 5.5% for class-switched HA-specific Bmem. We found no significant correlation between *IGHV* SHM and AvIn values (Fig. S4), consistent with previous observations (20, 21).

10.1128/mbio.02546-22.4 FIG S4 SHM for non–class-switched and class-switched Bmem. (A) *IGHV* SHM of tetanus toxin (TT)-reactive Bmem. Median values are represented by horizontal lines, and mean values are included above each dataset for TT-specific non–class-switched and class-switched Bmem. (B and C) Avidity index versus SHM level of HA-specific Bmem. Correlation plots for infant 1 (B) and infant 2 (C) are shown for HA-specific non–class-switched and class-switched Bmem. Red, blue, and purple dots represent IAV-specific, IBV-specific, and IAV+IBV Bmem, respectively. Spearman correlation was nonsignificant (*P > *0.05) among all subsets. Download FIG S4, PDF file, 0.3 MB. Copyright © 2022 Kuraoka et al. 2022 Kuraoka et al. https://creativecommons.org/licenses/by/4.0/ This content is distributed under the terms of the Creative Commons Attribution 4.0 International license.

Because both infants received DTaP (diphtheria, tetanus, and pertussis) vaccinations concurrently with their year 2 IIV4 immunizations (Fig. 1A), we used tetanus toxoid (TT) as a control in our panel for the multiplexed bead assay and to compare SHM levels in TT-specific Bmem as an independent measure of the infants’ response to repeated (2 or 3) immunizations with a single immunogen. The overall frequencies of TT-reactive Bmem (Table S3) were similar for the two infants; the average SHM levels (e.g., 4.0% and 7.1% in infant 1 and infant 2, respectively, for year 2 postvaccination) (Fig. S4) were similar in each infant to the HA-specific SHM levels, even after 4 DTaP doses in the case of infant 2 (Fig. 1A).

10.1128/mbio.02546-22.7 TABLE S3 Frequencies of HA- and TT-reactive Bmem. Download Table S3, PDF file, 0.05 MB. Copyright © 2022 Kuraoka et al. 2022 Kuraoka et al. https://creativecommons.org/licenses/by/4.0/ This content is distributed under the terms of the Creative Commons Attribution 4.0 International license.

### HA-specific plasma antibody repertoire.

**FIG 4:**
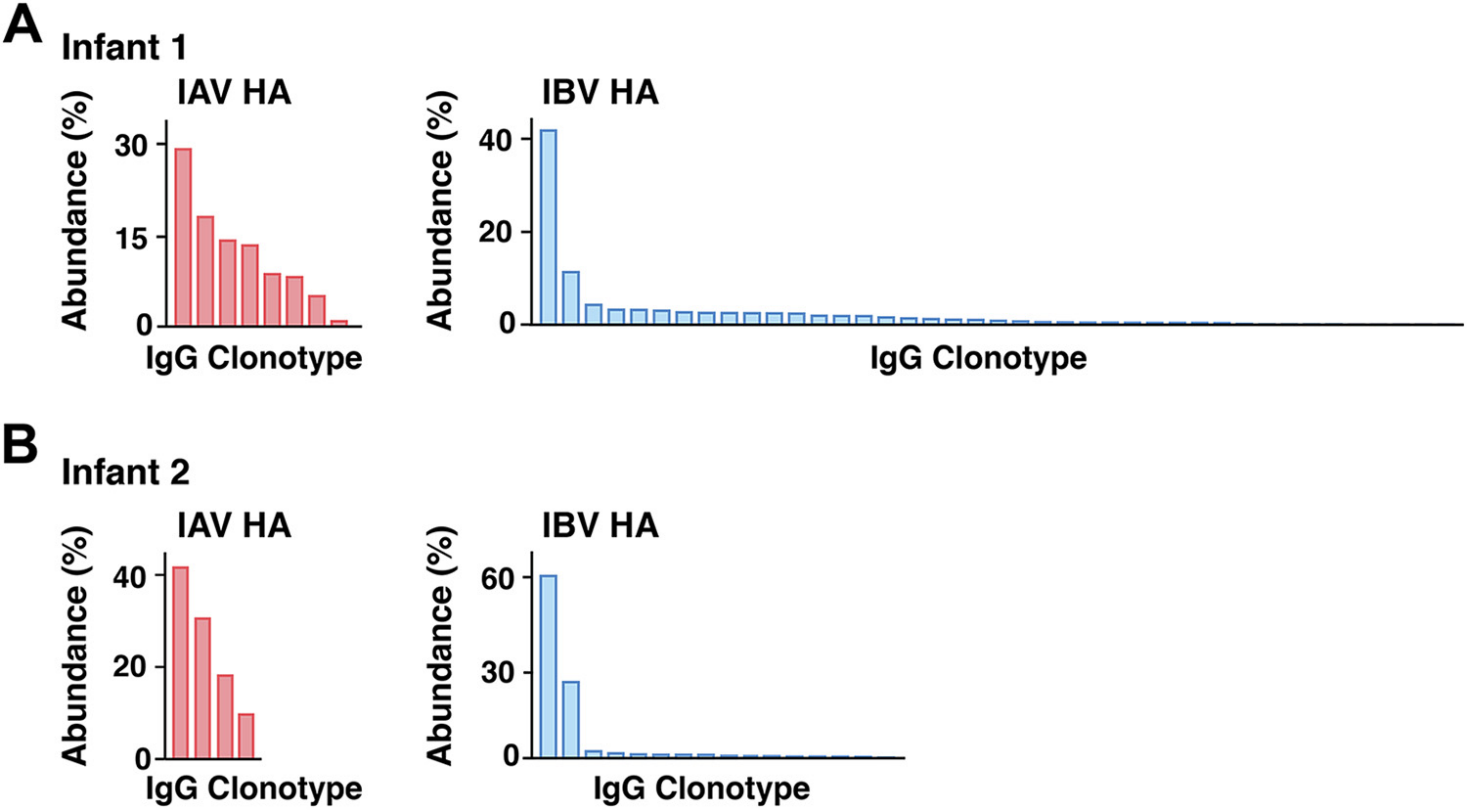
Summary of IAV and IBV HA-reactive plasma IgG repertoire. (A and B) Compositions of IAV and IBV HA-reactive plasma IgG antibody repertoires of infant 1 (A) and infant 2 (B). Each bar represents a unique antibody clonotype, and their heights correspond to the relative abundance of each clonotype in the IAV or IBV HA-reactive IgG repertoire.

Ig-Seq (9–11, 16) profiling of the IAV and IBV HA-reactive plasma IgG repertoires from the year 2 postvaccination time point (Data Set S2) showed that the serological antibody repertoires of both infants were oligoclonal, with 8 (infant 1) and 4 (infant 2) IAV HA-reactive clonotypes and 41 (infant 1) and 16 (infant 2) IBV HA-reactive clonotypes (Fig. 4). With the exception of the IBV HA-specific repertoire of infant 1, the total number of distinct antibody clonotypes specific for each HA was much smaller than the numbers previously observed in adults, which ranged between 40 and 147 at different postvaccination time points (11). In both infants, one highly abundant clonotype dominated the IBV HA-specific repertoire, comprising 42% and 61% of the entire repertoire in infants 1 and 2, respectively; the third most abundant clonotype accounted for only 4.3% and 2.3% of the repertoires (infant 1 and infant 2, respectively). The distribution of the IAV HA-specific repertoire was less polarized.

10.1128/mbio.02546-22.10 DATA SET S2 Identities, relative abundances, and molecular features of individual antibody clonotypes in the serological repertoire of IAV- and IBV-reactive antibodies from each infant, as analyzed by Ig-Seq. Download Data Set S2, XLSX file, 0.02 MB. Copyright © 2022 Kuraoka et al. 2022 Kuraoka et al. https://creativecommons.org/licenses/by/4.0/ This content is distributed under the terms of the Creative Commons Attribution 4.0 International license.

The most abundant IBV HA-specific plasma IgG clonotype from infant 1 was clonally related to a year 2 postvaccination Bmem BCR that bound trimeric HA (but not the head alone) with high avidity (AvIn of ~0.2) and broad specificity for B/Phuket/2013, B/Florida/2017, and B/Brisbane/2008 and 3.4% SHM (Data Set S1). The second most abundant clonotype was clonally related to a year 2 prevaccination Bmem BCR with high avidity (AvIn of ~0.7) for B/Phuket/2013. The 3.4% SHM level of this clone, like that of the third most abundant, both presumably affinity matured in response to the IBV infection, was considerably higher than the SHM levels of the most abundant clonotypes in the IAV HA-specific repertoires of both infants 1 and 2 (1.7% and 1.4%) and in the IBV HA-specific repertoire of infant 2 (0.9%) (Data Set S2).

## DISCUSSION

Technologies like single-cell NGS and Ig-Seq have enabled detailed molecular dissection of human immune responses to vaccination and infection (11, 16). Before widespread implementation of infant influenza vaccination, most children had experienced an IAV or IBV infection by the age of 3 or 4 (22); previous studies characterizing immune responses to later vaccinations in adults were therefore in the context of preexisting immune memory. The principal challenge in extending such studies to true primary responses in infants is how to extract as much information as possible from the small volumes of blood that can be drawn. Because of restricted blood draw volumes from infants (no more than 0.7 mL/kg for infants and children, compared to 40 to 50 mL typically drawn from adult subjects), we maximized the depth of BCR repertoire analysis by combining NGS and Nojima culture expansion and sequencing (Fig. 1B). We also performed one affinity purification step using mixed IAV HAs and another using mixed IBV HAs to define the IAV and IBV HA-reactive plasma IgG repertoires, respectively. The results presented here illustrate that our approach can lead to meaningful conclusions, despite the limited sampling.

The SHM levels of the overall Bmem repertoires in the infants studied here reflect both affinity maturation in response to the many vaccines in the standard series and environmental exposure to antigens that children commonly encounter. They conform to patterns previously documented for the overall development of B cell repertoires (23–26). For example, a recent examination of the immunological characteristics of childhood B cell development (16) found that by age 3, infants had adult levels of SHM in non–class-switched B cells and levels in class-switched B cells that approached the 7 to 8% typical of adults (15). Our data show, specifically for HA, that the range of SHM levels in Bmem 2 to 3 weeks after the year 2 boost was likewise comparable to the range for similar vaccine responses in adults after multiple antigen exposures by vaccination and infection. The specific response to influenza that we have analyzed gives a closer view of affinity maturation of HA-reactive antibodies after successive boosts of a particular antigen from vaccination and infection.

For infant 1, IBV infection shifted the Bmem repertoire with an AvIn above the background level from primarily IAV HA reactive in year 1 to substantially IBV directed in year 2 (Fig. 3A and B). The year 2 vaccination retained and boosted this IBV-biased Bmem repertoire. To the extent that the IBV infection occurred in a nearly IBV-naive background, the single immunization having generated undetectable numbers of Bmem, imprinting by infection (IBV) apparently resulted in a type-dominated response, partly eclipsing whatever IAV imprint the year 1 vaccination had produced. For infant 2, the year 2 vaccination probably retained the IAV/IBV balance of the primary response, but the total number of B cells retrieved in the pooled 2- and 3-week postvaccination samples (Table S3) was too modest to support a strong conclusion.

For both infants, the average SHM levels after the initial, year 1 vaccination (IIV4 no. 1), 2.3% and 2.7%, respectively, were similar to those (upper level of ca. 2.5%) generally found for primary responses to vaccination with a novel antigen (27–30). The IBV infection of infant 1 may have produced more extended affinity maturation—a mean of 4% SHM prevaccination in year 2—although we cannot rule out that some of the Bmem isolated at that time point were descendants of cells elicited by the year 1 immunization. There is evidence for relatively long-lived germinal centers (several months) following infection with severe acute respiratory syndrome coronavirus 2 (SARS-CoV-2) (31–34) and correspondingly higher SHM levels at the end of the active response. The class-switched, IBV HA-specific Bmem isolated from infant 1 after the year 2 immunization had avidities similar to the average avidity of adult influenza HA-specific BCRs, indicating that the Bmem outcome of infection followed by a single vaccination boost is nearly indistinguishable in SHM and AvIn values from the responses of adults imprinted by infection but vaccinated (and perhaps infected) multiple times since then.

Plasma antibodies 2 to 3 weeks after a boost reflect the plasmablast burst (which peaks at about 1 week) derived from reactivated Bmem differentiating into antibody-secreting cells in the background of contributions of LLPCs from all previous exposures. The plasma antibody repertoires in the two infants we studied were more restricted than those in adult donors and dominated by an individual clonotype in the IBV-reactive plasma IgG. It remains undetermined how the repertoire develops in subsequent years and in response to subsequent exposures. Studying the course of immunity in a larger number of subjects and over longer times will be necessary to determine how typical are the differences we have found here and, if they are indeed more general, whether subsequent exposures modify their respective characteristics.

## MATERIALS AND METHODS

### Study approvals and volunteers.

The study procedures, informed consent, and data collection documents were reviewed and approved by the Duke Health IRB (Pro00020561, initial approval 2009). We enrolled two infants who were vaccinated during their first year and boosted a year later (Table S1). From infant 1, blood was collected on days 0 and 13 following IIV4 no. 1 in year 1 and days 0, 14, and 21 following IIV4 no. 2 in year 2. From infant 2, blood was collected on days 0 and 14 following IIV4 no. 1 in year 1 and days 0, 14, and 21 following IIV4 no. 3 in year 2. Blood samples were processed into PBMCs and plasma and then aliquoted and stored at −150°C for future analysis.

### Flow cytometry.

PBMCs in RPMI medium containing 10% fetal bovine serum (FBS) were incubated with excess mouse IgG1 (MG1K; Rockland) to block nonspecific binding and then labeled with fluorochrome-conjugated monoclonal antibodies (MAbs). The following human surface antigen-specific MAbs, purchased from BD Biosciences, BioLegend, or Thermo Scientific, were used: anti-human IgM-fluorescein isothiocyanate (FITC) (MHM-88), CD3-phycoerythrin (PE)-Cy5 (UCHT1), CD14-Tri (TuK4), CD16-PE-Cy5 (3G8), CD19-PE-Cy7 (HIB19), IgG-allophycocyanin (APC) (G18-145), IgD-PE (IA6-2), CD27-BV421 (M-T271), and CD24-BV510 (ML5) antibodies. Labeled cells were sorted by using the FACSAria II with Diva software (BD Biosciences). Flow cytometric data were analyzed with FlowJo software (Tree Star, Inc.). Total Bmem (CD19^+^ Dump^−^ CD27^+^ CD24^+^) and IgG^+^, IgG^−^, and IgM^+^ Bmem (Fig. S2) were identified as previously described (18, 19, 35). Doublets were excluded from cell sorting by combinations of forward scatter area (FSC-A) versus FSC height (FSC-H) gating. Cells positive for 7-aminoactinomycin D (7-AAD) (BD Bioscience) or for CD3, CD14, or CD16 expression were also excluded.

### Single-cell cultures.

Single Bmem were expanded in the presence of MS40L-low feeder cells as previously described (18, 19, 36). Single Bmem were sorted directly into wells of 96-well plates and cocultured for 25 days with the MS40L-low feeder cells (37, 38) in the presence of exogenously added cytokines. Half of the culture medium was replaced twice each week with fresh medium containing fresh cytokines. On day 25, culture supernatants were harvested for screening the reactivity of clonal IgG antibodies. Expanded clonal B cells were frozen at −80°C for V(D)J sequence analyses.

### BCR rearrangement amplification and analysis.

Rearranged V(D)J gene sequences for human B cells from single-cell cultures were obtained by SMART (switching mechanism at 5′ end of RNA template) reverse transcription (RT)-PCR. Total RNA was extracted from selected samples using the Quick-RNA 96 kit (Zymo Research). cDNA was synthesized from DNase I-treated RNA using SMARTScribe reverse transcriptase (Clontech) at 42°C for 90 min, followed by 70°C for 15 min, with 0.2 μM each gene-specific reverse primer (hIgG-RV1, hIgKC-RV1, and hIgLC-RV2) and 1 μM 5′ SMART template-switching oligonucleotide (TSO-bioG). The primers used for RT were as follows: hIgG-RV1, GGTGTTGCTGGGCTTGTGA; hIgKC-RV1, CCTGTTGAAGCTCTTTGTGAC; hIgLC-RV2, GTGCTCCCTTCATGCGTGA; and TSO-bioG, 5Biosg-CCAAGCTG GCTAGCACCATGACAGrGrGrG, which contains biotin at its 5′ end (5Biosg) and three riboguanosines (rGrGrG) at its 3′ end.

The synthesized cDNA was then subjected to SMART PCR using Herculase II Fusion DNA polymerase (Agilent Technologies) with a common forward primer (5Anchor1-FW1, CAAGCTGGCTAGCACCATGA) and gene-specific reverse primers (hIgG-RV2 [GGTCACCACGCTGCTGA], hIgKC-RV3 [GCTGTAGGTGCTGTCCTTG], and hIgLC-RV3 [TGTGGGACTTCCACTGCTC] for IgG and kappa and lambda region PCR, respectively). The PCR conditions were 95°C for 4 min, followed by 30 cycles at 95°C for 30 s, 55°C for 20 s, and 72°C for 30 s. The PCR products were submitted to the Duke DNA sequencing facility to obtain DNA sequences. The primers used for sequencing were hIgG-RV4 [GGGAAGTAGTCCTTGACCAG], hIgKC-RV4 [CTGGGAGTTACCCGATTGGA], and hIgLC-RV4 [GCTGGCCGCRTACTTGTTG] for IgG and kappa and lambda regions, respectively.

### V_H_:V_L_ paired sequencing.

Bmem collected 13 days after IIV4 no. 1 from infant 1 and B cells collected 14 days after IIV4 no. 3 from infant 2 were analyzed by V_H_:V_L_ paired sequencing as previously described (39, 40). Single B cells were encapsulated in emulsion droplets in a custom flow-focusing apparatus. The droplets contained lysis buffer and poly(dT)-conjugated magnetic beads to capture mRNA from single B cells. The magnetic beads with mRNA were collected and emulsified to undergo overlap extension RT-PCR, in which V_H_ and V_L_ transcripts were physically linked and amplified by reverse transcription xenopolymerase (RTX) (41). V_H_:V_L_ amplicons were extracted, amplified by nested PCR, and sequenced using an Illumina MiSeq (2 × 300-bp paired-end reads).

### HA expression and purification.

Recombinant HA (rHA) head and rHA full-length soluble ectodomain (FLsE) constructs were cloned into the pFastBac vector for insect cell expression (Hi5 cells) as previously reported (6, 42–45). All constructs were confirmed by DNA sequencing at the DNA Sequencing Core Facility at Dana Farber Cancer Institute. The HA FLsE constructs used in ELISAs contained a thrombin or human rhinovirus (HRV) 3C protease-cleavable C-terminal foldon tag with either a 6×His or streptavidin-binding peptide (SBP)-8×His tag. All constructs were purified from supernatants by passage over Co-nitrilotriacetic acid (NTA) TALON resin (TaKaRa) followed by size exclusion chromatography on a Superdex 200 Increase column (GE Healthcare) in 10 mM Tris-HCl, 150 mM NaCl at pH 7.5. Affinity tags were removed using HRV 3C protease (Thermo Scientific), and the protein repurified using Co-NTA TALON resin to remove the protease, tag, and noncleaved protein.

### Multiplex bead assay.

The specificities and avidities of clonal IgG antibodies in culture supernatants were determined in a multiplex bead assay (Luminex Corp.) as described previously (18, 36), with modifications. Culture supernatants were serially diluted in 1× phosphate-buffered saline (PBS) containing 1% bovine serum albumin (BSA), 0.05% NaN_3_, and 0.05% Tween 20 (assay buffer) with 1% milk and incubated for 2 h with the mixture of antigen-coupled microsphere beads in 96-well filter-bottom plates (Millipore). After washing three times with assay buffer, the beads were incubated for 1 h with PE-conjugated mouse anti-human IgG antibodies (JDC-10; Southern Biotech). After three washes, the beads were resuspended in assay buffer and the plates read on a Bio-Plex 3D suspension array system (Bio-Rad). Antigens and controls included BSA, mouse anti-human Ig(κ) (SB81a; Southern Biotech), mouse anti-human Ig(λ) (JDC-12; Southern Biotech), mouse anti-human IgG (Jackson ImmunoResearch), streptavidin (Invitrogen), tetanus toxoid from Clostridium tetani (List Biological Laboratories), NP (4-hydroxy-3-nitrophenylacetyl hapten)-BSA (coupled in the Kelsoe laboratory), keyhole limpet hemocyanin (KLH; Sigma), ovalbumin (OVA; Siga), kynureninase (KYNU; Sino Biological), recombinant protective antigen (rPA; BEI Resources), HIV-1 gp140 JR-FL, mutant KYNU (provided by the Duke Human Vaccine Institute) (46), insulin (Sigma), and a panel of recombinant hemagglutinins (full-length constructs of H1/California/2009 X181, H1/Michigan/2015 X-275 [vaccine strain], H1/Michigan/2015 [circulating strain], H1/Florida/1993, H1/Singapore/2015 IVR 180, H3/Hong Kong/2014 X263B [vaccine strain], H3/Hong Kong/2014 [circulating strain], B/Phuket/2013, B/Florida/2017, and B/Brisbane/2008 and head constructs of H3/Hong Kong/2014 X263B, B/Phuket/2013, B/Florida/2017, and B/Brisbane/2008). A control MAb (CR9114) was used to check for any biases in the HAs used (Table S4).

10.1128/mbio.02546-22.8 TABLE S4 Mean fluorescence intensity (MFI) values for CR9114 binding to each HA used in Luminex assay. Download Table S4, PDF file, 0.04 MB. Copyright © 2022 Kuraoka et al. 2022 Kuraoka et al. https://creativecommons.org/licenses/by/4.0/ This content is distributed under the terms of the Creative Commons Attribution 4.0 International license.

### Plasma antibody titers.

Binding to recombinant HA proteins was performed as described previously (47). High-binding 384-well microtiter plates were coated with recombinant HA protein at a final concentration of 2 μg/mL diluted in 0.1 M NaHCO_3_ and incubated overnight at 4°C. The plates were washed with 1× PBS containing 0.1% Tween 20 and blocked for 1 h with blocking buffer (40 g whey protein, 150 mL goat plasma, 5 mL Tween 20, 0.5 g NaN_3_, 40 mL 25× PBS, brought up to 1 L with water). The plates were washed, and 10 μL of diluted plasma (starting at 1:30 and serially diluted in blocking buffer) added directly to each well, followed by incubation for 1.5 h. The plates were washed and then incubated for 1 h in blocking buffer without NaN_3_ and with horseradish peroxidase (HRP)-conjugated secondary antibody goat anti-human IgG Fc-specific antibody (Jackson ImmunoResearch), added at the dilution recommended by the manufacturer. The plates were washed and developed with 3′,5-tetramethylbenzidine substrate (KPL) for 15 min. Development was stopped with 1% HCl (Fisher Scientific). The plates were read on a plate reader (Molecular Devices) at 450 nm. The background for each analyte was determined based on nonimmune plasma. Midpoint (50% effective dose [ED_50_]) titers were calculated by applying four-parameter logistic regression to the binding data using the *drc* package in R (48).

### Purification of total IgG from plasma and subsequent digestion into F(ab′)2.

Plasma samples collected following IIV4 no. 2 (day 14, 130 μL; day 21, 500 μL) from infant 1 and following IIV4 no. 3 (day 14, 250 μL; day 21, 500 μL) from infant 2 were passed through a 1.5-mL protein G agarose (Pierce) affinity column three times in gravity mode. The column was washed with 20 column volumes of Dulbecco’s PBS (DPBS) and eluted with 10 mL of 100 mM glycine-HCl (pH 2.7), and the eluate was immediately neutralized with 1.5 mL of 1 M Tris-HCl (pH 8.0). Purified IgG was digested into F(ab′)2 with 200 μg of IdeS per 10 mg of IgG for 6 h on a thermal mixer (Eppendorf ThermoMixer C) at 37°C. The digest was rotated with 200 μL of Strep-Tactin Superflow resin (IBA Lifesciences) at room temperature for 1 h and separated using a Pierce spin column, collecting the F(ab′)2 in the flowthrough.

### Antigen enrichment of F(ab′)2 and mass spectrometry (MS) sample preparation.

rHAs were immobilized on separate columns for the antigen-specific F(ab′)2 enrichment as previously described (11). Briefly, rHAs were separately immobilized on *N*-hydroxysuccinimide (NHS)-activated agarose resins (Pierce) by overnight rotation at 4°C; 0.5 mg of mixed H1/Michigan/2015 X-275 and H3/Hong Kong/2014 X263B or mixed B/Brisbane/2008 and B/Phuket/2013 (vaccine strains) were used for infant 1 and 0.5 mg of mixed H1/Michigan/2015 X-275 and A/H3/Singapore/2016 or B/Maryland/2016 and B/Phuket/3073/2013 (vaccine strains) were used for infant 2 to generate two affinity resins per infant. The coupled agarose resins were washed with DPBS, and unreacted NHS groups were blocked with 1 M Tris, pH 8, for 30 min at room temperature. The resins were further washed with 5 mL of DPBS and added to F(ab′)2 samples. Resin and F(ab′)2 mixtures were rotated for 1 h at room temperature. Flow-through samples were collected, and the columns were washed with 10 column volumes of DPBS. Antigen-enriched F(ab′)2 was eluted with 1% formic acid in 0.50 mL. Flow-through and elution fractions were assayed by indirect ELISA with the donor-specific rHAs, and the depletion of ELISA signal in each flow-through sample evaluated. Elution fractions showing an optical density at 450 nm (OD_450_) of >0.1 were pooled and concentrated under vacuum to a volume of <10 μL, resuspended in 40 μL of PBS, and neutralized with 1 M NaOH.

For each enrichment, elution and flow-through samples were denatured in 50 μL of 2,2,2-trifluoroethanol (TFE) and 5 μL of 100 mM dithiothreitol (DTT) at 55°C for 45 min and then alkylated by incubation with 3 μL of 550 mM iodoacetamide (Sigma) for 30 min at room temperature in the dark. Samples were diluted 10-fold with 40 mM Tris (pH 8) and digested with trypsin (1:30 trypsin/protein) for 12 h at 37°C. Formic acid was added to 1% (vol/vol) to quench the digestion, and the sample volume was reduced to ~100 μL under vacuum. Peptides were then bound to a C18 HyperSep SpinTip extraction tip (Thermo Fisher Scientific), washed three times with 0.1% formic acid, and eluted with 60% acetonitrile, 0.1% formic acid. The C18 eluate was dried under vacuum centrifugation and resuspended in 50 μL of 5% acetonitrile, 0.1% formic acid.

### LC-MS/MS.

Samples were analyzed by liquid chromatography-tandem mass spectrometry (LC-MS/MS) on either a Dionex Ultimate 3000 RSLCnano ultra-high performance liquid chromatography (UHPLC) system (Thermo Fisher Scientific) or an Easy-nLC 1200 (Thermo Fisher Scientific) coupled to an Orbitrap Fusion Tribrid or Velos pro mass spectrometer (Thermo Fisher Scientific). Peptides were first loaded onto either an Acclaim PepMap RSLC nano trap column (Dionex; Thermo Fisher Scientific) or, prior to separation, on a 75-μm by 15-cm Acclaim PepMap rapid-separation liquid chromatography (RSLC) C18 column (Dionex; Thermo Fisher Scientific) using either a 5-to-40% (vol/vol) or 5-to-32% (vol/vol) acetonitrile gradient over 95 or 120 min at 300 nL/min. The eluting peptides were injected directly into the mass spectrometer using a nano-electrospray source. The instruments were operated in a data-dependent mode with parent ion scans (MS1) collected at 120,000 or 60,000 resolution. Monoisotopic precursor selection and charge state screening were enabled. Ions with a charge of +2 or greater were selected for collision-induced dissociation fragmentation spectrum acquisition (MS2) in the ion trap. Sample were run three times to generate technical-replicate data sets.

### MS/MS data analysis.

Donor-specific protein sequence databases were constructed using the donor’s corresponding V_H_ sequences with ≥2 reads, concatenated to a database of background proteins comprising non–donor-derived V_L_ sequences (HD1) (9), a consensus human protein database (Ensembl 73, longest sequence per gene), and a list of common protein contaminants (MaxQuant). Donor-specific spectra were searched against the corresponding donor-specific databases using SEQUEST HT (Proteome Discoverer 2.4; Thermo Fisher Scientific). The searches considered fully tryptic-digested peptides only, allowing up to two missed cleavages. A precursor mass tolerance of 5 ppm and a fragment mass tolerance of 0.5 Da were used. Modifications of carbamidomethyl cysteine (static) and oxidized methionine (dynamic) were selected. High-confidence peptide spectrum matches (PSMs) were filtered at a false discovery rate of <1% as calculated by Percolator (q-value of <0.01) (Proteome Discoverer 2.4; Thermo Fisher Scientific).

Iso/Leu sequence variants were collapsed into single peptide groups. For each scan, PSMs were ranked first by posterior error probability (PEP), then q-value, and finally based on cross-correlation (XCorr). Only unambiguous top-ranked PSMs were kept; scans with multiple top-ranked PSMs (equivalent PEP, q-value, and XCorr) were designated ambiguous identifications and removed. The average mass deviation (AMD) for each peptide was calculated as described previously (10) using data from all injections, and peptides with an AMD of >1.7 ppm were removed.

Peptide abundance was calculated from the extracted-ion chromatogram (XIC) peak area as described previously (9), using peak area values generated by the Precursor Ions Area Detector node in Proteome Discoverer. For each peptide, a total XIC area was calculated as the sum of all unique XIC areas of associated precursor ions, and the average XIC area across replicate injections was calculated for each sample. The peptide PSM was calculated as the sum of the aforementioned PSMs. For each antigen data set, the eluate and flow-through abundances were compared, and peptides with a ≥5-fold-higher signal in the elution sample were considered to be antigen specific.

### Clonotype indexing and peptide-to-clonotype mapping.

V_H_ sequences were grouped into clonotypes based on single-linkage hierarchical clustering as described previously (9, 11). Cluster membership required ≥90% identity across the CDR-H3 amino acid sequence as measured by Levenshtein distance. High-confidence peptides identified by MS/MS analysis were mapped to clonotype clusters, and peptides uniquely mapping to a single clonotype were considered “informative.” The abundance of each antibody clonotype was calculated by summing the XIC areas of the informative peptides mapping to ≥4 amino acids of the CDR-H3 region (i.e., CDR-H3 peptides). For each antibody clonotype, the most abundantly detected CDR-H3 peptide (in cases where peptides from multiple somatic variants belonging to the same clonotype were detected) was used as a representative CDR-H3 sequence.

Clonotype abundance was calculated as the sum of the peptide abundances belonging to a clonotype. When multiple nonoverlapping tryptic peptides were available for a clonotype, one peptide was selected and used for the analysis to prevent double counting and both the PSMs and XICs were summed across the collapsed peptides.

### Recombinant antibody synthesis, expression, and purification.

The sequences of variable heavy- and light-chain regions for the chosen antibodies were codon optimized and synthesized as eBlocks (Integrated DNA Technologies). The synthetic genes were cloned into a pcDNA3.4 vector (Invitrogen) containing human IgG1 heavy-chain and kappa or lambda light-chain constant regions, respectively (16, 49). Heavy- and light-chain plasmids for each monoclonal antibody were cotransfected into Expi293F cells (Thermo Fisher Scientific). After incubating for 5 days at 37°C with 8% CO_2_, the supernatant containing secreted antibodies was centrifuged at 4,000 × *g* for 15 min at 4°C, filtered through 0.45-μm syringe filters (Sartorius), and passed three times over a column with 1 mL protein G agarose resin (Pierce). After washing the column with 20 column volumes of PBS, antibodies were eluted with 10 mL 100 mM glycine-HCl (pH 2.7) and immediately neutralized with 1.5 mL of 1 M Tris-HCl (pH 8.0). Antibodies were buffer exchanged into DPBS, utilizing Amicon ultra-30 centrifugal spin columns (Millipore).

### ELISA.

Ninety-six-well EIA/RIA assay microplates (Corning) were coated with 4 mg/mL antigen at 4°C overnight. Plates were washed with PBST, blocked with 2% BSA in PBS for 2 h at room temperature, and washed again. Serially diluted recombinant antibodies or plasmas were added to the plates and incubated for 1 h. The plates were washed, and 1:5,000-diluted goat anti-human IgG Fc HRP-conjugated secondary antibodies (Invitrogen) were added. After a 1-h incubation, plates were washed and 100 mL ultra TMB (tetramethylbenzidine) substrate (Thermo Fisher Scientific) added to each well to develop the signal; 50 mL 2 M H_2_SO_4_ was added to quench the reaction. Absorbance was measured at 450 nm (OD_450_) with a SpectraMax i3x plate reader. Data were analyzed and fitted for 50% effective concentration (EC_50_) if necessary, using a four-parameter logistic nonlinear regression model in the GraphPad Prism (9.0) software.

### Statistical analysis.

Statistical analyses were performed using GraphPad Prism 9.0 (GraphPad Software, Inc., San Diego, CA). All statistical tests performed are described in the figure legends, and significance was determined by a *P* value of ≤0.05.

### Data availability.

The nucleotide sequences for heavy- and light-chain variable domains of the antibodies listed in Data Set S1 have been deposited in GenBank with accession numbers OP419769 to OP419958. All the Ig-Seq data (raw and processed proteomics data sets, along with the reference sequence databases) from this study have been uploaded to MassIVE and can be accessed under the identifier MSV000090057.
