# Supplementary material for: Infant Antibody Repertoires during the First Two Years of Influenza Vaccination
Source: mBio. 2022 Oct 31;13(6):e02546-22. doi: 10.1128/mbio.02546-22 (PMC9765176; doi:10.1128/mbio.02546-22)
Supplement: TABLE S1 [file mbio.02546-22-s0005.pdf]

Table S1. IIV immunizations and their components

Timing of IIV4 immunizations for infants 1 and 2

Infant	Birth Year	Age at Year 1 IIV4	Adminstered IIV4	Age at Year 2 IIV4	Adiminstered IIV4
Infant 1	2016	7 months	2016-2017 IIV4	17 months	2017-2018 IIV4
Infant 2	2016	12 months	2017-2018 IIV4	25 months	2018-2019 IIV4

HA components of IIV4 in the seasons relevant to this study

	IIV4 Components			
Year	A/H1 Component	A/H3 Component	B/Victoria Component	B/Yamagata Component
2016-2017	H1/California/2009 X-181	H3/Hong Kong/2014 X-263B	B/Brisbane/60/2008	B/Phuket/3073/2013
2017-2018	H1/Michigan/2015 X-275	H3/Hong Kong/2014 X-263B	B/Brisbane/60/2008	B/Phuket/3073/2013
2018-2019	H1/Michigan/2015 X-275	H3/Singapore/2016 IVR-186	B/Maryland/15/2016	B/Phuket/3073/2013
