# Supplementary material for: Infant Antibody Repertoires during the First Two Years of Influenza Vaccination
Source: mBio. 2022 Oct 31;13(6):e02546-22. doi: 10.1128/mbio.02546-22 (PMC9765176; doi:10.1128/mbio.02546-22)
Supplement: FIG S1 [file mbio.02546-22-s0001.pdf]

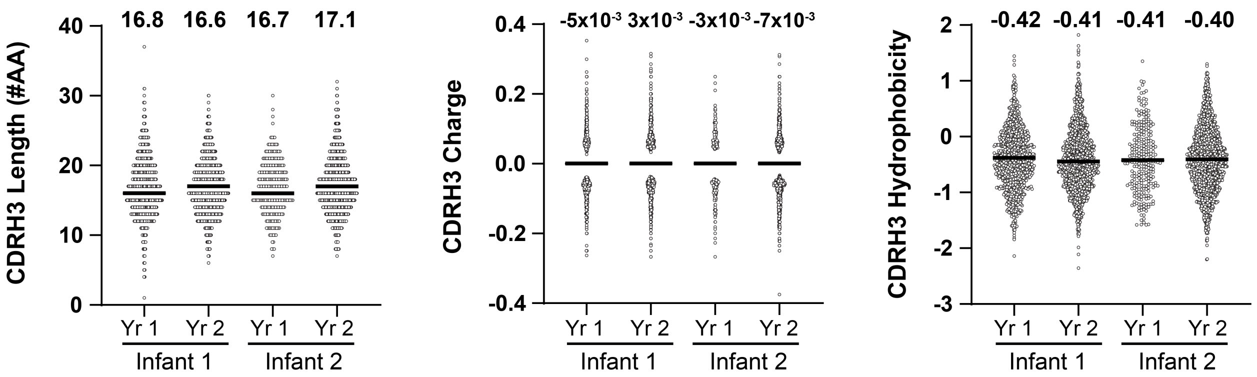

Fig. S1. CDRH3 length, charge, and hydrophobicity of total post-vaccination B cells. Median values are represented by horizontal lines and mean values are included above each dataset.
