# Supplementary figures and images for: Infant Antibody Repertoires during the First Two Years of Influenza Vaccination
Source: mBio. 2022 Oct 31;13(6):e02546-22. doi: 10.1128/mbio.02546-22 (PMC9765176; doi:10.1128/mbio.02546-22)

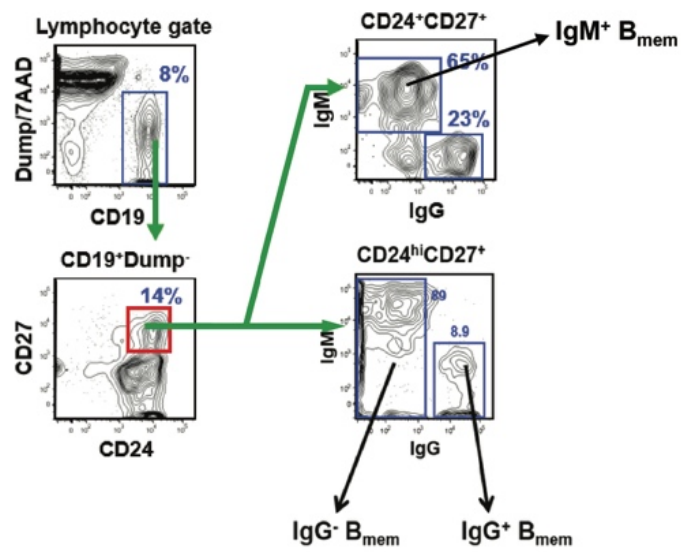

Fig. S2. Representative sorting strategy to isolate B<sub>mem</sub> for Nojima culturing.

Supplement: FIG S2 [file mbio.02546-22-s0002.pdf]
