# Supplementary material for: Infant Antibody Repertoires during the First Two Years of Influenza Vaccination
Source: mBio. 2022 Oct 31;13(6):e02546-22. doi: 10.1128/mbio.02546-22 (PMC9765176; doi:10.1128/mbio.02546-22)
Supplement: FIG S3 [file mbio.02546-22-s0003.pdf]

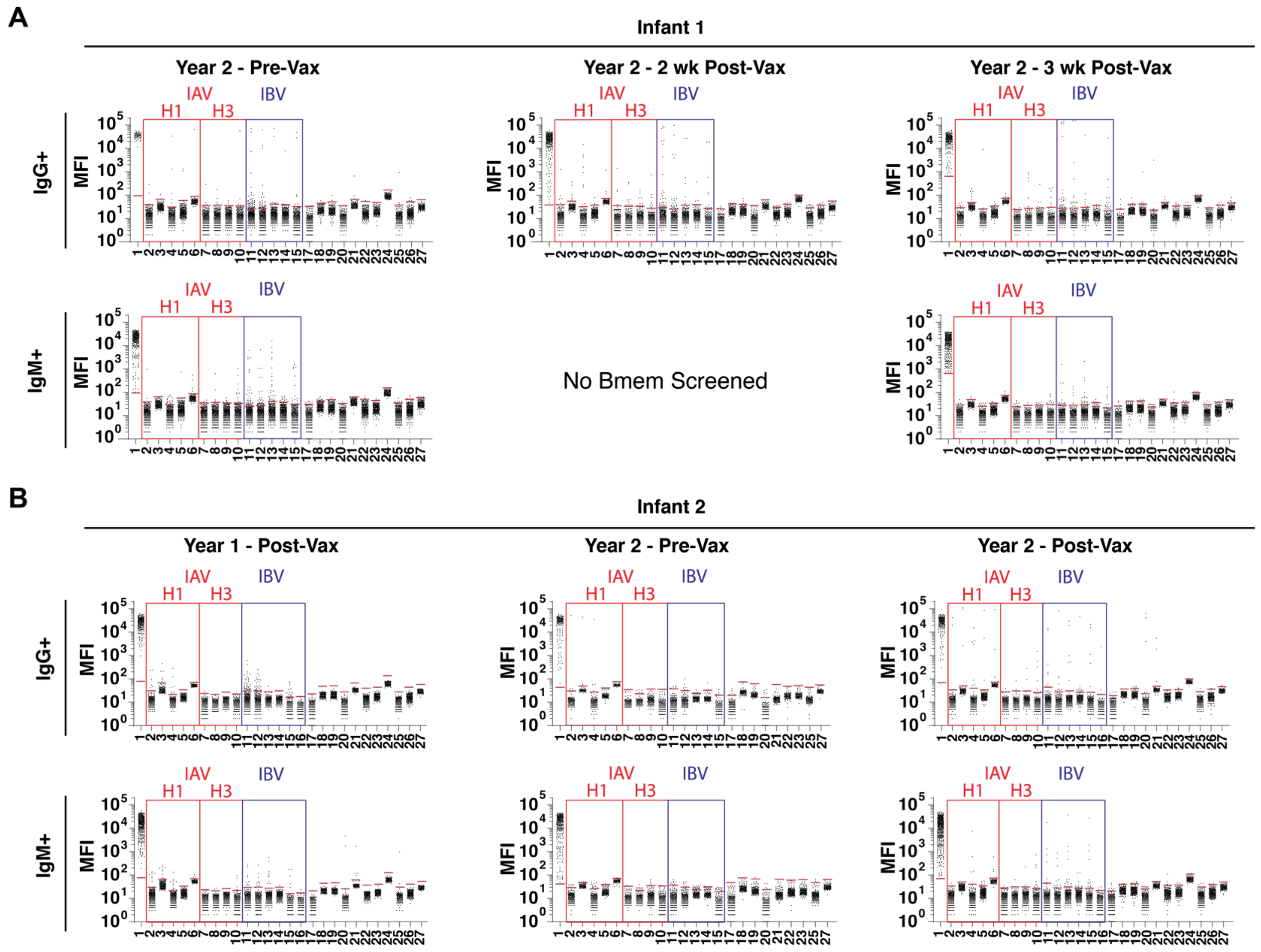

ID	Antigen
1	IgG
2	CA-09 X-181
3	MI-15
4	MI-15 X-275
5	FL-93
6	SI-06
7	HK-14 263b
8	HK-14 263b-H
9	HK-14
10	HK-68 X-31
11	Phu-13
12	Phu-13-H
13	FL-17
14	FL-17-H
15	Bris-08
16	Bris-08-H
17	BSA
18	Streptavidin
19	gp140 JR-FL
20	Tetanus Toxoid
21	NP-BSA
22	KLH
23	OVA
24	KYNU
25	rPA
26	mutKYNU
27	Insulin

Fig. S3. Luminex reactivity assays. Red lines indicate background measurements for each antigen. List on the right shows antigen corresponding to each numbered column. See Materials and Methods (Multiplex Bead Assay) for full name of each HA and control antigen. IgG: mouse anti-human IgG. Antigens marked H are trimeric, head-only constructs.
