# Supplementary material for: Infant Antibody Repertoires during the First Two Years of Influenza Vaccination
Source: mBio. 2022 Oct 31;13(6):e02546-22. doi: 10.1128/mbio.02546-22 (PMC9765176; doi:10.1128/mbio.02546-22)
Supplement: TABLE S2 [file mbio.02546-22-s0006.pdf]

Table S2. Recombinant mAbs from Infant 1 Year 1 post-vaccination Bmem. Clonotypes with high read counts from the NGS data were made as mAbs. For each selected clonotype, the CDRH3 sequence, *IGHV*, *IGHJ*, and SHM levels are shown. mAbs were characterized by ELISA to determine their EC_{50} values against HAs. +++: <100 nM; ++: 100-1000 nM; +: detected signal at 1000 nM; -: no signal at 1000nM; H1: A/California/2009 X181; H3: A/Hong Kong/2014 X263B; B/Vic: B/Brisbane/60/2008; B/Yama: B/Phuket/3073/2013.

Description	CDRH3	*IGHV*	*IGHJ*	*IGHV* SHM (%)	H1 Binding	H3 Binding	B/Vic Binding	B/Yama Binding
IgG-rank 1	CAKDFYGSWGSMDVW	3-9	6	0.7	-	-	-	-
IgG-rank 2	CARGNGPGSYLIDYW	1-3	4	6.4	+	-	-	-
IgG-rank 3	CARGGGTMIVPIKPHNWYFDLW	1-18	2	1.7	+++	++	-	-
IgG-rank 4	CARVRPLTVGWHFDLW	4-61	2	0.7	+++	++	-	-
IgG-rank 5	CARDGLKVGTTTFDYW	3-66	4	1.4	+++	++	-	-
IgG-rank 6	CARGGGPYYYGSGSYYNSLYYYYYYMDVW	4-34	6	1	+	+	-	-
IgG-rank 7	CAKDSPSGWYKGGFDYW	3-30	4	4.9	-	-	-	-
IgG-rank 8	CARNGGGLGLW	3-74	3	5.7	+	+	-	-
IgG-rank 9	CARDWEYSSSFFGPW	3-11	5	3.7	+	+	-	-
IgG-rank 11	CARESFKGNWFDPW	3-53	5	5.5	+++	+++	-	-
IgG-rank 14	CAREIIAVADPTDGARNWYFDLW	3-53	2	2.1	+++	++	-	-
IgM-rank 1	CARGAQTTVTTLVDYYYYYYMDVW	4-34	6	0	++	-	-	-
IgM-rank 2	CNTGSVAAAEPW	3-15	5	2.4	+	+	-	-
IgM-rank 3	CARDQLVHDAFDIW	4-31	3	0	++	+	-	-
IgM-rank 4	CARDTLNCGGDCYGDYW	3-21	4	2.1	+++	++	-	-
IgM-rank 5	CARDRDYGYFDYW	1-3	4	1.7	+	-	-	-
