# Supplementary material for: Infant Antibody Repertoires during the First Two Years of Influenza Vaccination
Source: mBio. 2022 Oct 31;13(6):e02546-22. doi: 10.1128/mbio.02546-22 (PMC9765176; doi:10.1128/mbio.02546-22)
Supplement: FIG S4 [file mbio.02546-22-s0004.pdf]

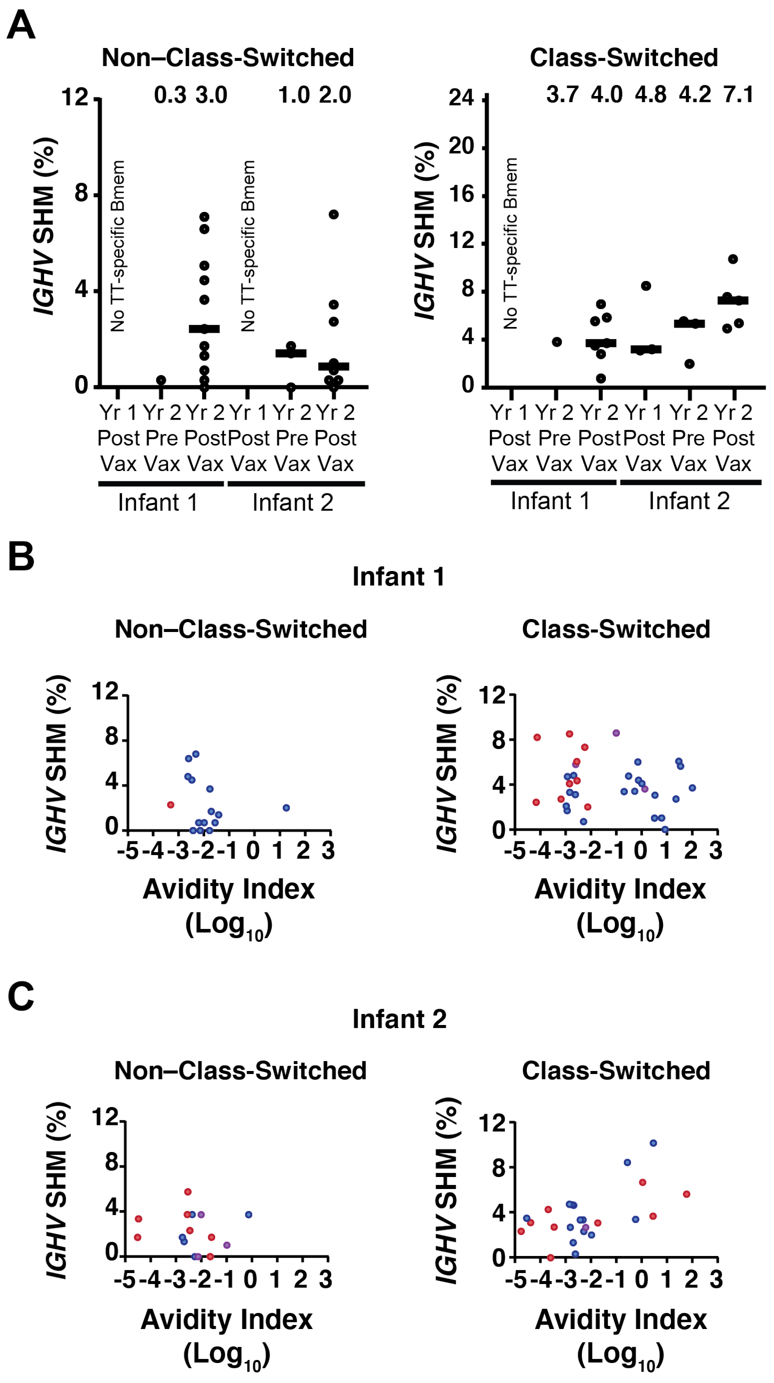

Fig. S4. SHM for non-class-switched and class-switched Bmem. (A) *IGHV* SHM of tetanus-toxin (TT) reactive Bmem. Median values are represented by horizontal lines and mean values are included above each dataset for TT-specific non–class-switched and class-switched Bmem. **(B and C) Avidity index vs. SHM level of HA-specific Bmem.** Correlation plots for Infant 1 (**B**) and Infant 2 (**C**) are shown for HA-specific non–class-switched and class-switched Bmem. Red, blue, and purple dots represent IAV-specific, IBV-specific, and IAV+IBV Bmem, respectively. Spearman correlation was non-significant ($P > 0.05$) among all subsets.
