# Supplementary material for: Infant Antibody Repertoires during the First Two Years of Influenza Vaccination
Source: mBio. 2022 Oct 31;13(6):e02546-22. doi: 10.1128/mbio.02546-22 (PMC9765176; doi:10.1128/mbio.02546-22)
Supplement: TABLE S3 [file mbio.02546-22-s0007.pdf]

Table S3: Frequency of HA- and TT-reactive Bmem.

	Infant 1		Infant 2	
	Yr 2 Pre-Vax	Yr 2 Post-Vax	Yr 2 Pre-Vax	Yr 2 Post-Vax
IAV	4/1422 (0.28%)	6/1505 (0.40%)	1/694 (0.14%)	3/1169 (0.26%)
IBV	18/1422 (1.27%)	17/1505 (1.13%)	1/694 (0.14%)	5/1169 (0.43%)
IAV+IBV	1/1422 (0.07%)	2/1505 (0.13%)	0/694 (0.00%)	4/1169 (0.34%)
Tetanus	2/1422 (0.14%)	21/1505 (1.40%)	6/694 (0.86%)	14/1169 (1.20%)
