# Supplementary material for: Infant Antibody Repertoires during the First Two Years of Influenza Vaccination
Source: mBio. 2022 Oct 31;13(6):e02546-22. doi: 10.1128/mbio.02546-22 (PMC9765176; doi:10.1128/mbio.02546-22)
Supplement: TABLE S4 [file mbio.02546-22-s0008.pdf]

Table S4: MFI values for CR9114 binding to each HA used in Luminex assay.

	HAs	Luminex MFI Values
H1	CA-09 X-181	2.73E+04
	MI-15 X-275	8.64E+04
	SI-06	6.31E+04
H3	HK-14 263b	5.55E+03
	HK-68 X-31	1.75E+04
B	Phu-13	1.31E+04
	FL-17	9.12E+03
	Bris-08	1.29E+04
